# Two cases of primary ovarian neuroblastoma arising from mature cystic teratomas^☆^

**DOI:** 10.1016/j.gynor.2013.04.006

**Published:** 2013-05-07

**Authors:** Yuri Niwa, Osamu Yamamuro, Noriko Kato, Toyonori Tsuzuki

**Affiliations:** a Department of Obstetrics and Gynecology, Nagoya Daini Red Cross Hospital, Japan; b Department of Pathology, Nagoya Daini Red Cross Hospital, Japan

**Keywords:** Primary ovarian neuroblastoma, Mature cystic teratoma origin, Prognosis, Chemotherapy

## Abstract

• Primary ovarian neuroblastomas are extremely rare and have poor prognoses.
• This report provides results of the same combination therapy in two cases, which indicated its efficacy for ovarian neuroblastoma.

## Introduction

Neuroblastoma accounts for approximately 8–10% of all tumors in children aged 15 years, and the incidence of neuroblastomas 1 in 7000 in the United States.

Neuroblastoma treatment is based on the clinical stage and categories. Surgery and/or chemotherapy are recommended. Most infants with disseminated disease have a favorable outcome after chemotherapy and surgery, while the majority of children aged > 18 months, who have advanced neuroblastoma, die of progressive disease despite intensive multimodality therapy.

Neuroblastomas develop from neural crest cells, and their anatomical locations are essentially the adrenal glands and paraspinal sites. Only a few reports of primary ovarian neuroblastoma have been reported worldwide. Although it is commonly known that malignancy occurs in 0.3–4.8% of mature cystic teratomas of the ovary (Mori et al., 2003), neurobalstomas arising from mature cystic teratomas are extremely rare, as we found only 4 cases published in the literature since 1982 (Reid et al., 1983; Kleinman et al., 1993; Kiyozuka et al., 1994; Muhlstein et al., 2010). Almost all ovarian neuroblastomas have very poor prognoses; however, we performed intensive treatment with a combination of surgery and chemotherapy and found no occurrence of relapse in two cases. Particularly, case 1 achieved complete remission for > 13 years in spite of multiple metastases.

## Case Reports

### Case 1

**Fig. 1:**
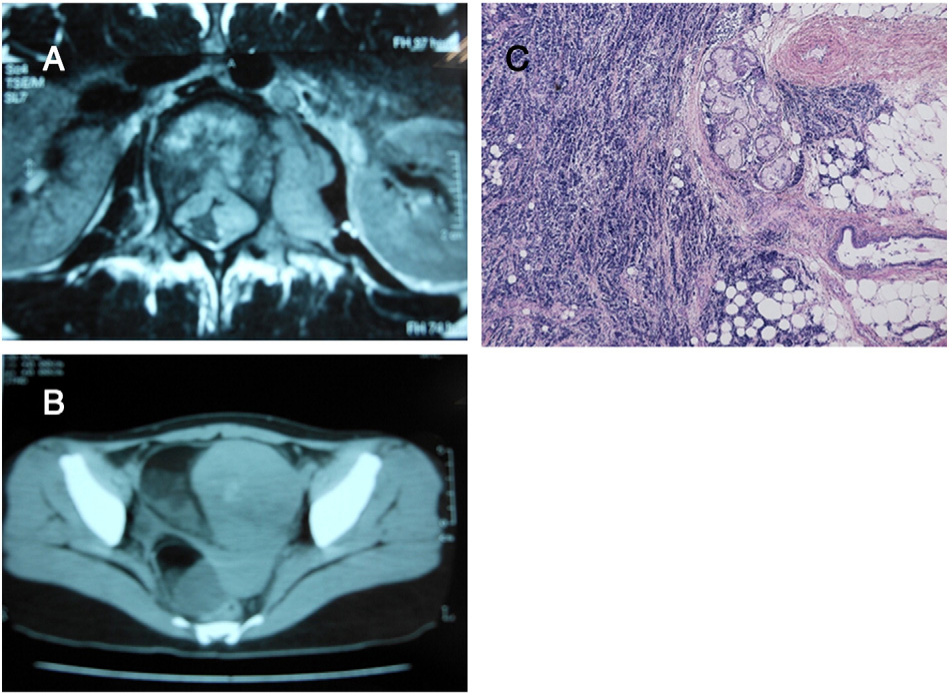
Imaging and microscopic findings (case 1). A: Paravertebral tumor compressed spinal canal. B: Pelvic CT scan showed bilateral ovarian tumors. C: Histological diagnosis of neuroblastoma (left side) and mature cystic teratoma (right side).

A 22-year-old Japanese woman presented with difficulty in walking and numbness of her right thigh. It was revealed by magnetic resonance imaging (MRI) that she had a paravertebral tumor from the first to third lumbar vertebrae (Fig. 1A). A computed tomography (CT) scan showed bilateral ovarian tumors (Fig. 1B). Paravertebral tumor resection was performed immediately. Because the aim of surgery was spinal decompression, many tumors remained. Because her platelet count was low, bone marrow aspiration was performed on the iliac crest, as metastasis was suspected. Bone scintigraphy showed Th6, L1, L2, right ilium, left femur, right rib metastases. The serum level of neuron specific enolase (NSE) was 57 ng/mL, ferritin was 1520 ng/mL, and lactate dehydrogenase (LDH) was 1652 IU/L. Approximately 2 weeks after vertebra surgery, we performed left salpingo-oophorectomy and right ovarian tumor resection. These tumors were resectable.

Microscopically, two different tumor components were observed: a neuroblastoma and a mature cystic teratoma (Fig. 1C), which were connected. Therefore, we diagnosed this patient with primary ovarian neuroblastoma. The right ovary revealed a mature cystic teratoma. The serum levels of vanillylmandelic acid (VMA) and homovanillic acid (HVA) were 1.0 and 1.3 mg/L, respectively. We began triweekly intravenous chemotherapy with a combination of etoposide (100 mg/m^2^ on days 1–5) and cisplatin (20 mg/m^2^on days1–5). A major complication was grade2 myelosuppression that was effectively managed with granulocyte colony-stimulating factor (G-CSF). The residual tumors disappeared as indicated by CT, MRI, and bone scintigraphy after completion of a course of 6 treatments. The serum levels of NSE, ferritin, and LDH returned to normal, and, to date, no relapse has been observed over a 13-year follow-up period.

### Case 2

**Fig. 2:**
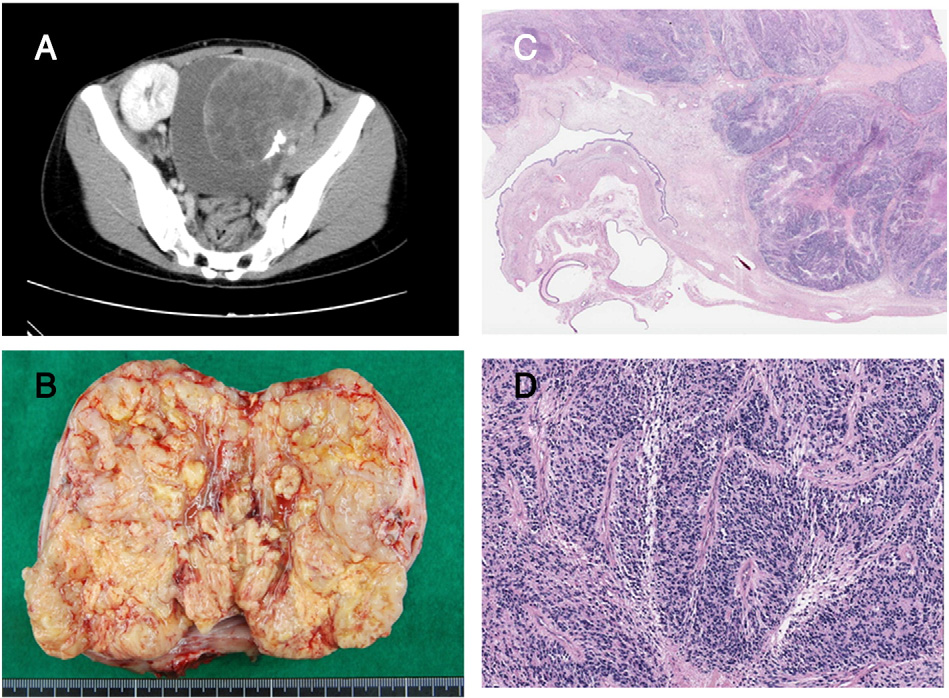
Imaging and microscopic findings (case 2). A: Pelvic CT scan revealed a 120 × 115 × 125 mm mass. B: Gross finding. C: Histological diagnosis of neuroblastoma (right side) and mature cystic teratoma (left side). D: Neuroblastoma (high magnification).

A 25-year-old Japanese woman complained of abdominal bloating and received a living donor (her mother) kidney transplant for chronic renal failure due to renal hypoplasia when she was 13 years old. A pelvic CT scan revealed a 120 × 115 × 125 mm mass (Fig. 2A). The tumor markers were as follows: cancer antigen (CA)-125, 51 U/mL; CA-72-4, 4.6 U/mL; and squamous cell carcinoma antigen,2.0 ng/mL. Laparotomy was performed. It revealed that the patient's right ovary was increased in size, although her uterus, fallopian tubes, and left ovary had normal appearances. Right salpingo-oophorectomy and harvest of ascites was performed. We considered that there was no gross residual disease. The patient's postoperative course was uneventful, and the final pathology report indicated neuroblastoma arising from a mature cystic teratoma (Fig. 2C,D). Postoperative bone scintigraphy was performed. It revealed no bone metastasis. Twenty-four-hour urine samples were collected postoperatively. These samples showed that the urinary excretion concentrations of VMA and HVA were normal. Ascites cytology was negative. Thus, we diagnosed International Federation of Gynecology and Obstetrics stage 1C(b) because of intraoperative microrupture due to adhesion. Therefore, we administered adjuvant chemotherapy with etoposide and cisplatin. Dose reductions were necessary to protect renal function. Thus, we decreased the etoposide dose by 25% (75 mg/m^2^ on days 1–5) and cisplatin by 50% (10 mg/m^2^on days 1–5) every 3 weeks. Her renal function was good, and we used G-CSF to treat grade2 myelosuppression. She remained in good health with no evidence of recurrence for 6 months after 6 courses of chemotherapy.

## Discussion

Neuroblastomas can arise anywhere throughout the sympathetic nervous system, although the adrenal glands are the most common primary site. There have been only 9 cases of primary ovarian neuroblastoma reported worldwide since 1982 (Reid et al., 1983; Kleinman et al., 1993; Kiyozuka et al., 1994; Muhlstein et al., 2010; Aguirre and Scully, 1982; Block et al., 1984; Lawlor et al., 1997).

In our cases, the neuroblastomas likely arose from the neural tissue of mature cystic teratomas. In the past reports, 4 of 9 cases were associated with mature cystic teratomas or immature teratomas, although those with unclear origins have also been reported (Aguirre and Scully, 1982; Block et al., 1984; Lawlor et al., 1997). Mature cystic teratoma shared 10–20% of its pathology with that of the benign ovarian cyst and malignant change occurs in 0.3–4.8% (Mori et al., 2003). Although most malignancies are due to squamous cell carcinomas, changes in neuroblastomas are extremely rare.

Mori et al. (2003) reported that the average age of a patient with malignant transformation is 54.7 years, which is 17 years older than the average age of patients with mature cystic teratomas, whereas Peterson (1957) reported an average age of 45.5 years. These data suggest that malignant transformation may arise from mature cystic teratomas after an average of 10 years. On the other hand, Kiyozuka et al. (1994) reported that the average age of patients with primary ovarian neuroblastomas was 23.4 years, which was younger than those with common malignant transformations. Interestingly, neuroblastomas are common in infants and children, as the mean age at diagnosis is 2 years, and 90% cases occur before the age of 5 years. Malignant transformation to a neuroblastoma may occur earlier than that to the other epithelial carcinomas.

The prognosis of primary ovarian neuroblastoma is unfavorable. According to Muhlstein et al. (2010), in the 9 reported cases, including 4 malignant transformed teratoma cases, 4 patients died 2–7 months after operation, 2 patients relapsed 11 and 24 months after operation, and 3 patients achieved complete remission during the 7- to 24-month follow-up periods (Muhlstein et al., 2010). However, the reported remissions have an insufficient follow-up period, with a maximum of 2 years. At the time of this report, our patient achieved complete remission, which has been > 13 years after diagnosis of a primary tumor in spite of multiple metastases. Although the prognosis of ovarian neuroblastoma is unfavorable, some patients achieve complete remission in spite of multiple metastases, which is similar to infant neuroblastoma, as most infants with disseminated diseases have favorable outcomes following chemotherapy and surgery. The age at presentation is an important prognostic factor in childhood neuroblastoma (London et al., 2005). Thus, we believe that in cases with good prognosis, ovarian neuroblastomas develop subsequently from pre-existing mature cystic teratomas that arise in adults, whereas in cases with bad prognosis, ovarian neuroblastomas develop gradually from mature cystic teratomas.

Moreover, to investigate other factors in ovarian neuroblastoma, including N-myc amplification, DNA ploidy, and gain or loss of certain chromosomes that are known as prognostic factors in childhood neuroblastoma cases, may offer new insight into the prognosis of ovarian neuroblastoma. The treatment of primary ovarian neuroblastomas has not received a consensus. Thus, we suggest intensive treatment consisting of a combination of surgery and intravenous chemotherapy of cisplatin (20 mg/m^2^ on days 1–5) and etoposide (100 mg/m^2^ on days 1–5) once every 3 weeks for a total of 6 courses. As a treatment basis, Kiyozuka et al. (1994) reported this same regimen and recommended these agents as key drugs against childhood neuroblastoma. Dose reduction was necessary to protect renal function in case 2, and there was no major side effects, except for grade 2 myelosuppression in both patients, who are alive and present no evidence of relapse. Specifically, the patient in case 1 has maintained complete remission for > 13 years in spite of multiple metastases.

This is the first report to describe the use of surgical resection combined with multiagent chemotherapy in ovarian neuroblastoma.

## Conflict of interest statement

The authors declare that there are no conflicts of interest.
